# Optimization of Culture Protocols to Isolate *Leptospira* spp. from Environmental Water, Field Investigation, and Identification of Factors Associated with the Presence of *Leptospira* spp. in the Environment

**DOI:** 10.3390/tropicalmed5020094

**Published:** 2020-06-05

**Authors:** Udomsak Narkkul, Janjira Thaipadungpanit, Prapaporn Srilohasin, Preeraya Singkhaimuk, Metawee Thongdee, Somjit Chaiwattanarungruengpaisan, Panadda Krairojananan, Wirichada Pan-ngum

**Affiliations:** 1 Department of Tropical Hygiene, Faculty of Tropical Medicine, Mahidol University, Bangkok 10400, Thailand; udomsak.nau@student.mahidol.edu; 2 Mahidol-Oxford Tropical Medicine Research Unit, Faculty of Tropical Medicine, Mahidol University, Bangkok 10400, Thailand; janjira.tha@mahidol.ac.th (J.T.); prapaporn.srl@mahidol.ac.th (P.S.); p.singkhaimuk@gmail.com (P.S.); 3 Department of Clinical Tropical Medicine, Faculty of Tropical Medicine, Mahidol University, Bangkok 10400, Thailand; 4 Office for Research and Development, Faculty of Medicine Siriraj Hospital, Mahidol University, Bangkok 10700, Thailand; 5 The Monitoring and Surveillance Center for Zoonotic Diseases in Wildlife and Exotic Animals, Faculty of Veterinary Science, Mahidol University, Nakhon Pathom 73170, Thailand; metawee.tho@mahidol.ac.th (M.T.); somjit.cha@mahidol.ac.th (S.C.); 6 Department of Entomology, Armed Forces Research Institute of Medical Sciences, Rajvithi Road, Bangkok 10400, Thailand; PanaddaK.fsn@afrims.org

**Keywords:** *Leptospira* spp., culture, environmental water, initial bacterial load, environment–animal interaction

## Abstract

The successful culture of *Leptospira* spp. from the environment is challenging. Here, we optimized the isolation of *Leptospira* spp. from water samples spiked with different species and initial concentrations of this organism. The time periods between water sampling and the isolation process were varied (0, 2, and 4 weeks). Bacterial cultures were observed under a microscope, and cultures were graded for cell density, weekly, for 12 weeks. Most pathogenic *Leptospira* spp. were difficult to culture under all conditions. All conditions of water samples spiked with novel species of *Leptospira* subclade P1 were culture positive within 2 weeks. For *Leptospira* subclade P2, storing samples for 2 weeks prior to isolation resulted in more successful isolation compared with isolation after other storage conditions. For subclade S1, all samples with initial bacterial concentrations of more than 10^3^ colonies/mL, under all storage conditions, were successfully cultured. These results suggest that storing contaminated water samples for 2 to 4 weeks in the dark at an ambient temperature prior to culturing can improve the isolation of *Leptospira* spp. from the samples. We implemented this protocol and collected water samples from natural sources accessed by both humans and animals. *Leptospira* spp. was identified in 32% (35/109) of water samples. The animal species using a water source influenced the likelihood of water samples being contaminated with *Leptospira* spp. Cultures of *Leptospira* spp. from environmental samples can provide useful information for understanding the complex interactions between humans, animals and the environment in the transmission of leptospirosis.

## 1. Introduction

Leptospirosis is a zoonotic, neglected tropical disease that occurs worldwide; it is caused by spirochete bacteria from the genus *Leptospira* [1,2]. The genus comprises more than 35 named species, divided into two major clades: Saprophytes (S) and pathogens (P). Recently, these two clades have been subdivided into a number of subclades, subclades P1 (pathogen group), P2 (intermediate group), S1 (saprophyte group), and S2 (a new subclade) [3]. Leptospirosis infection was first recognized as an occupational hazard for individuals working in agriculture, sewer maintenance, or animal husbandry and occurs as a result of direct or indirect contact with the urine of infected animals [2,4]. Other common modes of transmission include exposure to urine-contaminated water during recreational activities, adventure travel, and ingestion of contaminated water supplies [5,6,7,8]. People can become infected via wounds, mucous membranes such as the conjunctiva, and through wet skin [4]. 

Many leptospirosis outbreaks have been associated with water-related events, such as rural and urban flooding, swimming and other water sports, and occupational exposure linked predominantly to farming, as well as drinking contaminated water [7,8,9,10]. This suggests that the effective detection of *Leptospira* in the environment is important if adequate control measures are to be developed. Some studies suggest that *Leptospira* bacteria can adjust well to survive and persist under specific environmental conditions and moreover there is no evidence to suggest that pathogenic *Leptospira* bacteria lose their infectivity when they are in the environment [11].

Currently, the successful isolation of these organisms by culturing is limited due to the presence of non-pathogenic *Leptospira* spp. in the environment. In addition, the detection of pathogenic *Leptospira* spp. in water samples is difficult due to dilution of the pathogen in samples collected in the field, as well as the potentially high number of other bacterial species present in water samples, which can contaminate culture media. To date, there is no standard protocol for culturing the pathogenic species of *Leptospira* from environmental samples, in particular soil or water samples. Furthermore, there have only been a few reports that have described the isolation of pathogenic *Leptospira* spp. from environmental samples; as discussed above, the main difficulty involved in the isolation of these species is the overgrowth of other environmental bacteria or fast-growing saprophytic *Leptospira* spp. [12,13,14]. Whilst polymerase chain reaction (PCR) techniques have been developed to differentiate pathogenic from non-pathogenic *Leptospira* spp., protocols for testing for the presence of *Leptospira* spp. in water samples are in their preliminary stages; they have yet to be developed to the point where they are fully validated, universally accepted, or routinely performed [15,16,17,18,19]. Such tests are required to be both sensitive and specific in addition to being robust, non-labor intensive, and cheap to perform [20]. 

Previously, most laboratories have performed culturing of *Leptospira* spp. at a time of their convenience, following the collection of field samples. Here, we developed an optimal standard operating procedure to prepare field samples for the laboratory culturing of *Leptospira* spp., we then piloted and used the protocol in a field investigation. Improvements in sample storage and preparation prior to quantification under the microscope would greatly contribute to addressing gaps in our knowledge regarding the survival of *Leptospira* spp. in the environment. The findings from both our experiments and field investigation are presented here.

## 2. Materials and Methods 

### 2.1. Spiking Experiment

#### 2.1.1. Bacterial Strains and Water Samples Spiked with *Leptospira*

To investigate the effect of the length of water storage time on *Leptospira* isolation from water samples, six species of genus *Leptospira*, representing both pathogens and non-pathogens that live in the environment, were selected for the experiments. These included three species of subclade P1 isolated from blood samples from infected patients (*L. interrogans* strain L0013 [21] and *L. weilii* strain LNT1194 [22]); a putative novel species of pathogenic *Leptospira* from a natural water source (isolate PA62-C1, unpublished data); two species of subclade P2, isolated from human urine (*L. wolfii* strain H2 [23]) and flood water (*L. licerasiae* strain Q127 [24]); and one species of subclade S1 (*L. biflexa* strain LT17, received from the World Health Organization (WHO)/The Food and Agriculture Organization of the United Nations (FAO)/the World Organisation for Animal Health (OIE) Collaborating Centre for Reference and Research on Leptospirosis, Queensland, Australia). 

A 2 L sample of fresh water from a canal in Bangkok was collected and autoclaved at 121 °C for 15 min prior to use. All six strains of *Leptospira* were inoculated in Ellinghausen-McCullough-Johnson-Harris (EMJH) medium for 5 days until they reached the late-log phase of growth. Then, 100 mL water samples spiked with *Leptospira* culture were prepared as follows. Ten-fold serial dilutions of the bacterial culture (from 10^−1^ to 10^−7^) were prepared in a final volume of 20 mL, using the autoclaved canal water as a diluent. To prepare water samples containing approximately 10^2^ to 10^6^ colonies per mL, a 10 mL sample of each bacterial dilution was added to a 1 L beaker containing 90 mL of the sterile fresh water. A 0.2 mL sample of each bacterial dilution was taken to estimate the bacterial concentration in the water samples, based on counting colonies on the EMJH agar plates following incubation under ambient conditions for one month. The beakers were covered with sterile aluminum foil and stored at an ambient temperature (26–28 °C) in a dark cupboard for various periods of time, as described in the further isolation experiments below. To investigate the effect of time on bacterial isolation from water samples, the isolations were performed at three different time points: After 0, 2, and 4 weeks of water storage in the dark at an ambient temperature.

#### 2.1.2. *Leptospira* Isolation by Culture

To perform *Leptospira* isolation, 5 mL of water was collected using a 5 mL syringe. The water sample was passed through a sterile 0.2 μm filter (Sartorius AG, Gottingen, Germany). Then, 1 mL of filtrate was inoculated into a 5 mL tube containing 3 mL semi-solid EMJH media supplemented with 10% rabbit serum. Each water sample was tested in duplicate. The autoclaved canal water with no spiking of *Leptospira* was included as a negative control. The samples were incubated at 30 °C in a dry cabinet for 12 weeks. Bacterial growth was observed each week under dark field microscopy. A minimum density of 600 to 800 *Leptospira* cells per field was required before any further laboratory processes were undertaken [25]. A four-level density grading system for *Leptospira* cells present per field of view was defined, using the average number from ten microscopic fields of view at 200 times magnification, as follows: +1, *Leptospira* cells in less than 25% of the field of view; +2, *Leptospira* cells between 25% and 50% of the field of view; +3, *Leptospira* cells between 50% and 75% of the field of view; and +4, *Leptospira* cells in more than 75% of the field of view.

### 2.2. Pilot Testing 

After conducting the laboratory experiments, we repeated the protocol for water from a natural setting, using nine water samples collected from household consumable water sources. The water samples were collected in 50 mL tubes and transported to the laboratory under ambient conditions. Each sample was used under the three conditions, i.e., 0, 2, or 4 weeks storage in the dark at an ambient temperature, prior to being cultured.

### 2.3. Field Investigation 

**Figure 1:**
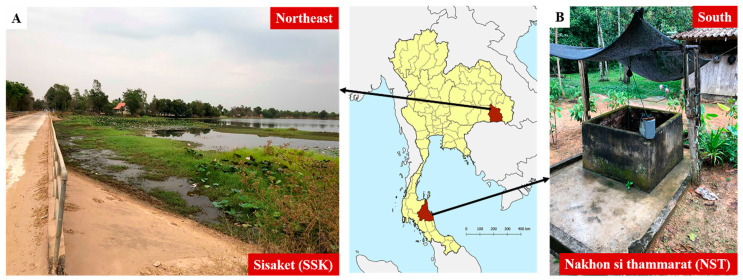
Typical shared water sources: (**A**) a natural water source with an open system, in the northeast; (**B**) a ground-water well owned by a household and situated close to or within the house, in the south.

We chose two provinces for our field investigations of environmental *Leptospira* spp.: Si Sa Ket (SSK) and Nakhon Si Thammarat (NST) provinces, in the northeast and south of Thailand, respectively. Relatively high rates of leptospirosis morbidity have been reported in these regions, according to national disease surveillance data and previous studies [26]. During the field investigation, we recorded key characteristics of water sources and the presence of different animal species around the water source in each of the sampling areas. Figure 1 shows the distinct features of typical water sources used in the northeast and the south of Thailand.

Samples were collected from environmental water sources surrounding households. Additional water samples were collected from ponds, ground water, tap water, rivers/canals, and other standing water sources that could be accessed by domestic animals (including cats and dogs), farm animals (including chickens, ducks, and pigs), cattle (including cows and buffaloes), rodents, and humans. The sample date, location, and volume of each sample was recorded. Samples were classified into one of four sample types: Pond, ground water, rivers or irrigation channels and tap water. A 50 mL centrifuge tube was filled with water from each water source. The tubes were transported to the laboratory under ambient conditions. Samples were separated in two; one 30 and one 20 mL sample. The 30 mL samples were stored for 2 weeks under ambient conditions until being processed for culturing [27], while the 20 mL samples were stored at −80 °C until being processed, using a direct PCR method targeting the 16S rRNA gene and lipL32 gene, as previously described [19]. Culture-positive samples were isolated.

### 2.4. Statistical Analysis

Statistical analysis was performed using Stata/SE 14.0 for Mac. The data were analyzed for both descriptive and inferential statistics. Independent categorical variables were described using frequencies and percentages. The outcome variable in our study, i.e., *Leptospira* contamination of a water source, was determined based on both culture and PCR results. Mixed-effects logistic regression was performed to identify risk factors associated with the outcome variable, after adjusting for all other variables. 

## 3. Results

### 3.1. Leptospira Isolation by Culture

Six *Leptospira* strains were used in the spiking experiment. For each strain, five different bacterial concentrations (10^2^ to 10^6^ colonies/mL) were individually spiked into the autoclaved water samples. The isolation process of these spiked water samples was performed over three different time periods: 0, 2, and 4 weeks of water storage in the dark at an ambient temperature. A total of 15 culture conditions for each strain tested were monitored for 12 weeks, as shown in Table 1.

For subclade P1, three strains were examined, including *L. interrogans* strain L13, *L. weilii* strain LNT1194, and the novel species strain PA62-C1. Only one condition of *L. interrogans* strain L13 was culture-positive: The maximum initial bacterial load, stored at an ambient temperature for 2 weeks prior to isolation. The observed duration for obtaining cultures of grade +3 or +4, i.e., a cell density sufficient for PCR processing, was 8 weeks. The cultures of water samples spiked with *L. weilii* strain LNT1194 were all negative regardless of the initial concentration, and prolonging water storage before culturing did not improve the isolation yield. Conversely, the presence of the novel species strain PA62-C1 was observed following all time periods and with any initial concentration. However, the lowest concentration (10^2^ colonies/mL) of PA62-C1 became positive only when the isolation was performed after samples had been stored for 2 or 4 weeks. The highest growth rate (reaching grade +4 or +3 within one week) was observed in cultures with the maximum initial bacterial concentrations only.

For subclade P2, we observed the presence of both tested strains, *L. wolffii* strain H2 and *L. licerasiae* strain Q127, with all time periods. *L. wolffii* strain H2 became positive at concentrations of 10^4^ colonies/mL or more. The isolation yields were one-log reduced when the isolation was performed immediately. The growth rates of spiked samples stored for 2 weeks were faster than those of samples stored for 4 weeks. *L. licerasiae* strain Q127, at any concentration, became positive in the culturing condition under which isolation was performed after being stored for 4 weeks. However, its growth rate was slower in cultures with lower concentrations (10^2^–10^3^ colonies/mL). The isolation yields were two- and three-log reduced when the spiked samples were processed immediately and after 2 weeks, respectively. 

For *L. biflexa* strain Patoc I, of subclade S1, the cultures were positive at a concentration of 10^5^ colonies/mL or more, irrespective of the storage period. Prolonging water storage did not increase the isolation yield. Unexpectedly, one was culture-positive at a concentration of 10^3^ colonies/mL after being stored for four weeks.

### 3.2. Leptospira Isolation in the Pilot Study

We repeated the experiments described above using nine natural water samples from household consumable water sources. The water samples were collected and placed in 50 mL tubes. Each sample was subjected to one of three conditions, i.e., storage for 0, 2, or 4 weeks in the dark at an ambient temperature, prior to being cultured. The results showed that no samples were positive immediately. One sample was positive for both the second and third conditions, i.e., isolation undertaken after water had been stored for 2 and 4 weeks, respectively. The positive sample was monitored each week for two more weeks before it reached a sufficiently high density (grade +3 or +4). This positive sample was confirmed by PCR to be a species in subclade P2, the intermediate group.

### 3.3. The Presence of Leptospira in Environmental Water in the Field 

Once our culture protocol had been optimized and tested using field samples, we applied this protocol by storing water samples for two weeks in the dark at an ambient temperature, prior to being cultured. There were 31 and 78 water samples collected in SSK and NST, respectively. Table 2 shows the percentage of positive samples obtained from culturing and/or direct PCR. There were 29% (9/31) and 33% (26/78) positive samples from SSK and NST, respectively. Positive samples were found in all consumable types of water including drinking water i.e., tap water in SSK and ground water in NST. Table 3 shows that among the 35 positive samples, 11 samples (31.4%), from waterfall and ground water in NST, were identified as pathogenic *Leptospira*. Furthermore, 11 samples (31.4%) were identified as intermediate *Leptospira*, these included two samples from tap water in SSK. In addition, sequencing and phylogenetic data analysis confirmed that the remaining 3 positive samples (8.6%) were non-pathogenic *Leptospira* and 10 positive samples (28.6%) were unculturable *Leptospira*, where there was insufficient PCR product for sequencing (data not shown).

### 3.4. Environment–Animal Interactions

We performed an analysis of the water source data collected in our study (n = 90). The presence of farm animals and domestic pets around water sources were the only two factors found to be associated with *Leptospira* contamination of the water sources; however, the association was in the opposite direction. The presence of farm animals around water sources was less likely to be linked with *Leptospira* contamination of water samples (adjusted odds ratio (AOR) 0.23, 95% confidence intervals (CI) 0.06–0.88), while the presence of domestic pets was 5.25 times (95% CI 1.53–18.03) more likely to result in *Leptospira* contamination of water samples (Table 4).

## 4. Discussion

As has been previously found, it was difficult to successfully culture pathogenic species of *Leptospira* and, if they could be cultured, they grew slowly. Previous studies have shown that pathogenic *Leptospira* spp. are able to survive but not multiply in the environment [28]. It is generally considered that pathogenic *Leptospira* spp. can survive in soil and bodies of fresh water, including mud, swamps, streams, lakes, and rivers, particularly under neutral to slightly alkaline conditions. Pathogenic *Leptospira* spp. have been noted to be highly susceptible to ultraviolet light, chlorine, and detergents. They are also thought to be susceptible to acidic conditions and low temperatures [28,29]. We successfully cultured one isolate of *L. interrogans* strain L13 under the conditions of maximum initial bacterial load and a time period from water sample collection to culture of 2 weeks. The growth rate of this species was quite slow (8 weeks to record a cell density grade of +4). None of the *L. weilii* strain LNT1194 samples were successfully cultured in our experiment. Conversely, the putative novel species of pathogenic *Leptospira* (isolate PA62-C1) was positive under all conditions, irrespective of the period of time between sampling and culturing and the initial bacterial load. The growth rate of the putative novel species was fast, taking just one week to reach a cell density grade of +4. Several recent studies from around the world have reported novel species of *Leptospira* from both animal and environmental (soil and water) samples [3,30,31], as well as in humans [32]. In Thailand, some novel species of pathogenic *Leptospira* were reported in floodwater in Bangkok during the 2011 floods [24] and in soil samples from southern Thailand. These isolates from southern Thailand were closely related to *L. ellisii* [33]; they did not induce signs or symptoms of leptospirosis in a hamster model infection [31]. These novel species tend to be well adapted to their environment and are able to survive culturing even when starting from a low initial concentration [3,31,32]. The experimental results we have described here confirmed this phenomenon.

For subclade P2, the experimental results for both species, *L. wolfii* strain H2 and *L. licerasiae* strain Q127, clearly showed that the optimal procedure for successful culturing was to store the isolates under ambient conditions in the dark for 4 weeks prior to the culturing process. This procedure was shown to increase the chance of obtaining positive cultures, especially for samples where the initial bacterial load was low. This finding may be explained by the bacteria requiring a period of time to adapt to their new environment and to be able to survive and grow [7]. 

For subclade S1, and *L. biflexa* strain Patoc I in particular, less than 50% of all isolates were successfully cultured. Only when the initial bacterial load was more than 10^4^ colonies/mL, was isolation of these organisms possible. Storing the samples for several weeks did not conclusively improve the ability to culture these organisms. This suggested that they are not good at penetrating the isolation filters and/or they do not survive well in water at high bacterial densities (10^5^–10^6^ colonies/mL). The latter reason is more likely and could explain why strain Patoc I, at a concentration of 10^3^ colonies/mL, became positive when subjected to isolation after 4 weeks of storage.

Generally, environmental water and soil are considered to be important reservoirs for leptospirosis transmission in humans and animals [11]. In our study, we tested 109 water samples from water sources used for consumption in the study areas. Around 30% of all samples were *Leptospira* spp. positive, either by culture or direct PCR. This indicated that a significant proportion of water sources contained *Leptospira*. The usual recommendations for avoiding *Leptospira* infection include wearing boots and thorough washing after work; however, this may not be sufficient given that both water sources around residential areas and those outside, in areas of work, may be contaminated [34,35]. In addition, the large differences observed between our study sites in terms of water contact patterns (including sharing of water sources) highlights the need for leptospirosis surveillance, prevention, and control strategies that take local environmental and behavioral factors into consideration.

Rodents have traditionally been the main animal reservoir of focus for the control of leptospirosis, although recently other animals have been considered as possible reservoirs of *Leptospira* spp. that have potentially contributed to human infections [36,37,38,39]. Research undertaken to investigate leptospirosis in dogs in Thailand has shown that certain behaviors in dogs may be associated with the prevalence of *Leptospira* antibodies [40], and pathogenic *Leptospira* spp. have been found to commonly occur in asymptomatic domestic animals [39]. Thus, in agreement with the findings of our association analysis, domestic pets, such as dogs, may be a significant contributor to the transmission of human leptospirosis. The presence of farm animals near to water sources, however, reduced *Leptospira* contamination of the water samples in our study. A previous study from Thailand also showed that pigs had the lowest seroprevalence among other potential reservoirs [36].

The main limitation of our study was the lack of data linking our findings to clinical cases of leptospirosis in humans in the same study areas, i.e., specific residential locations of leptospirosis cases and the timing of these infections. More precise information on these human infections could help determine appropriate locations and timing for environmental surveys.

## 5. Conclusions

The success of any isolation method for obtaining *Leptospira* spp. from water samples depends on the species, the strain characteristics, and the initial bacterial load. It is very difficult to culture pathogenic *Leptospira* spp. unless the initial bacterial load is high, i.e., at least 10^6^ colonies/mL. However, novel pathogenic *Leptospira* species and species from subclade P2, the intermediate group, appear to be highly adaptable and are able to grow well when present at concentrations of at least 10^3^ to 10^5^ colonies/mL, respectively. Extending the time period between collection and isolation can improve the isolation yield when a sample contains a lower initial bacterial load. In our study, we showed that storing contaminated water samples for 2 to 4 weeks in the dark at an ambient temperature prior to the culture process can improve *Leptospira* spp. isolation without being affected by the overgrowth of saprophytic or non-pathogenic *Leptospira* spp. Our study further identified the presence of domestic pets around shared water sources to be a risk factor associated with *Leptospira* spp. positivity in those water sources. These findings are useful for designing strategies to prevent environmental contamination with *Leptospira* spp. and to control human infections.

## Figures and Tables

**Table 1:** Isolation of *Leptospira* spp., where the growth of cultures was observed each week for 12 weeks. The shaded blocks represent the growth grades of *Leptospira* spp.

Strain				Conc. ^1^	Length of Storage Time (Weeks)																																			
					0												2												4											
Subclade P1	*L. interrogans*	L13		10^6^																																				
				10^5^																																				
				10^4^																																				
				10^3^																																				
				10^2^																																				
	*L. weilii*	LNT1194		10^6^																																				
				10^5^																																				
				10^4^																																				
				10^3^																																				
				10^2^																																				
		PA62-C1		10^6^																																				
				10^5^																																				
				10^4^																																				
				10^3^																																				
				10^2^																																				
Subclade P2	*L. wolfii*	H2		10^6^																																				
				10^5^																																				
				10^4^																																				
				10^3^																																				
				10^2^																																				
	*L. licerasiae*	Q127		10^6^																																				
				10^5^																																				
				10^4^																																				
				10^3^																																				
				10^2^																																				
Subclade S1	*L. biflexa*	Patoc I		10^6^																																				
				10^5^																																				
				10^4^																																				
				10^3^																																				
				10^2^																																				
Control																																								
**Weeks after culture**					1	2	3	4	5	6	7	8	9	10	11	12	1	2	3	4	5	6	7	8	9	10	11	12	1	2	3	4	5	6	7	8	9	10	11	12
0					+1									+2									+3									+4								

^1^ Initial bacterial load of *Leptospira* (colonies/mL of water).

**Table 2:** Laboratory results of water samples collected in this study and tested for *Leptospira* spp. * Samples were positive by either or both culture and direct PCR.

Type of Water	Percentage of Water Samples Suspected to be *Leptospira*-Positive					
	Sisaket (SSK) (n = 31)			Nakhon Si Thammarat (NST) (n = 78)		
	Culture	Direct PCR	Overall *	Culture	Direct PCR	Overall *
Puddle/pond/swamp	12.5% (3/24)	16.7% (4/24)	29.2% (7/24)	60.0% (3/5)	40.0% (2/5)	80.0% (4/5)
River/canal/waterfall	0% (0/5)	0% (0/5)	0% (0/5)	0% (0/3)	33.3% (1/3)	33.3% (1/3)
Tap water	100.0% (2/2)	0% (0/2)	100.0% (2/2)	-	-	-
Ground water	-	-	-	22.9% (16/70)	18.6% (13/70)	30.0% (21/70)
Total	16.1% (5/31)	12.9% (4/31)	29.0% (9/31)	24.4% (19/78)	20.5% (16/78)	33.3% (26/78)

**Table 3:** Classification of *Leptospira* spp. isolated from water samples collected in this study. * Samples were positive by either or both culture and direct PCR.

Type of Water	Percentage of Water Samples Positive for *Leptospira* * (n = 35)	
	Pathogenic *Leptospira*	Intermediate *Leptospira*
Puddle/pond/swamp	-	18.2% (2/11)
River/canal/waterfall	100% (1/1)	-
Tap water	-	100% (2/2)
Ground water	47.6% (10/21)	33.3% (7/21)
Total	31.4% (11/35)	31.4% (11/35)

**Table 4:** Factors associated with *Leptospira* contamination of water sources.

Characteristics	N ^1^ (%)	*Leptospira* Positive ^2^ (%)	OR ^3^ (95% CI)	AOR ^4^ (95% CI)
Presence of Rodents				
No	80 (88.89)	28 (35.00)	Reference	Reference
Yes	10 (11.11)	5 (50.00)	1.85 (0.49–6.96)	0.24 (0.02–2.64)
Presence of cattle				
No	73 (81.11)	24 (32.88)	Reference	Reference
Yes	17 (18.89)	9 (52.94)	2.29 (0.78–6.69)	3.09 (0.43–22.29)
**Presence of farm animals**				
**No**	**66 (73.33)**	**27 (81.82)**	**Reference**	**Reference**
**Yes**	**24 (26.67)**	**6 (18.18)**	**0.48** (**0.16**–**1.37**)	**0.23** (**0.06**–**0.88**)
**Presence of domestic pets**				
**No**	**67 (74.44)**	**20 (29.85)**	**Reference**	**Reference**
**Yes**	**23 (25.56)**	**13 (56.52)**	**3.05** **(1.15 –)**	**5.25** **(1.53 –18.03)**
Drinking source				
No	45 (50.00)	17 (37.78)	Reference	Reference
Yes	45 (50.00)	16 (35.56)	0.90 (0.38–2.14)	1.40 (0.40–4.87)
Consuming source ^5^				
No	37 (41.11)	17 (45.95)	Reference	Reference
Yes	53 (58.89)	16 (30.19)	0.05 (0.12–1.21)	0.37 (0.12–1.17)
Agricultural source				
No	58 (64.44)	18 (31.03)	Reference	Reference
Yes	32 (35.56)	15 (46.88)	1.96 (0.80–4.77)	1.56 (0.42–5.85)
Type of water source				
Closed system	85 (94.44)	31 (36.47)	Reference	Reference
Open system	5 (5.56)	2 (40.00)	1.16 (0.18–7.33)	1.34 (0.17–10.66)

^1^ Total sample of 90; ^2^ Total positive sample of 33; ^3^ Odds ratio (OR); ^4^ Adjusted odds ratio (AOR); ^5^ Consumable means using water in daily activities such as washing and cleaning.
